# Reply to Crimi, C.; Cortegiani, A. Comment on “Liu et al. Application of High-Flow Nasal Cannula in COVID-19: A Narrative Review. *Life* 2022, *12*, 1419”

**DOI:** 10.3390/life12101626

**Published:** 2022-10-18

**Authors:** Cheng-Wei Liu, Shih-Lung Cheng

**Affiliations:** 1 Department of Internal Medicine, Far Eastern Memorial Hospital, New Taipei City 22000, Taiwan; 2 Graduate Institute of Medicine, Yuan-Ze University, Taoyuan City 32003, Taiwan

Thanks for Crimi et al.’s comment [1]. The following is our response to your comment.

We recommend HFNC as a first-line treatment for patients with “severe” COVID-19 in the small discussion chapter of this review article. We are sorry for not giving clear definition of severe COVID-19 in our discussion chapter. The definition of severe COVID-19 here is to use 6–15 L O_2_/min (FiO_2_ 0.4–0.6) to achieve the target SpO_2_ (≥90% for nonpregnant patients and ≥92–95% for pregnant patients) [2]. In other words, HFNC may be suggested as a first-line treatment in patients with severe COVID-19 if COT (conventional oxygen therapy) can not meet adequate oxygenation in those patients, and IMV (invasive mechanical ventilation) is not indicated at the time. However, more well-designed RCTs in the future are needed to support this idea.

Indeed, a personalized and stepwise approach is important in treating COVID-19 patients with hypoxemic respiratory failure. Although there is still uncertainty about optimal oxygen strategy in COVID-19 patients with hypoxemic respiratory failure, HFNC may play an important role in a stepwise approach because of its great comfort for the patients.

We have learned much from your comment. Thank you again.
